# Comparison Study on Sonodirect and Sonoalternate Current Electrocoagulation Process for Domestic Wastewater Treatment

**DOI:** 10.1155/2022/3477995

**Published:** 2022-03-16

**Authors:** Lelisa Regea Mengistu, Zerihun Asmelash Samuel, Chali Dereje Kitila, Abreham Bekele Bayu

**Affiliations:** ^1^Jimma University, Jimma Institute of Technology, Faculty of Civil and Environmental Engineering, Jimma, Ethiopia; ^2^Jimma University, Jimma Institute of Technology, School of Chemical Engineering, Jimma, Ethiopia

## Abstract

Nowadays, there is a problem related to wastewater handling which is released from different activities. The electrocoagulation method has been a dominant treatment method for wastewater treatment. There are different forms of electrocoagulation methods for wastewater treatment. Nevertheless, there was no comparison made for the removal efficiency of the sonoalternate current (SAC), alternate current (AC), sonodirect current (SDC), and direct current (DC) electrocoagulation process. The efficiency of electrocoagulation methods was compared for their removal of chemical oxygen demand (COD) from Jimma University domestic wastewater. Batch Reactor DC/AC electrocoagulation cell was used to determine the removal efficiency. During the comparison, the response surface methodology (RSM) was used to analyze and optimize the data taken from the laboratory. Besides, ANOVA was used to analyze the interaction effects of different parameters. The removal of COD from domestic wastewater was achieved with DCE, ACE, SDCE, and SACE which were 82.6%, 86.58%, 88.6%, and 92.5%, respectively, under optimal experimental conditions. From the finding, SACE was more successful at removing % COD than the DCE, ACE, and SDCE methods. For DCE and SDCE, the formation of an impermeable oxide layer at the cathode and the occurrence of corrosion at the anode due to oxidation made the COD removal process less efficient compared with SACE processes. From the experimental results it can be concluded that the SACE has the lowest power consumption and higher process efficiency than the other EC methods and can be a promising solution for removing pollutants from domestic wastewater.

## 1. Introduction

Household wastewater is considered one of the world's leading sources of pollution [1]. Domestic wastewater is a dark brown liquid with high chemical oxygen demand (COD) and biochemical oxygen demand (BOD) due to a large number of organic substances such as proteins, polyphenols, organic acids, and polysaccharides [2]. Untreated wastewater from domestic wastewater can lead to high soil and water pollution [3–5]. However, the discharge of untreated domestic wastewater can lead to serious water pollution in both surface and groundwater, and increased concentrations of these pollutants pose a serious threat to flora and fauna, the environment, and humans [2, 6]. There has been increasing interest in recent years in finding creative ways to efficiently extract toxins from water, soil, and air [7]. Electrocoagulation and flotation are promising treatments based on electrochemical techniques [8].

Various chemical science processes such as back diffusion of chemical activity and natural biological effects designed wetland adsorption, pyrolysis, biological processes, and various new technologies such as advanced oxidation processes and membrane technologies [9], which are currently used for the treatment of household waste [10]. Chemical science methodologies are inefficient, require excessive use of chemicals, and produce large amounts of sludge [11]. At the same time, biological processing methodologies require a high degree of dilution, which is a slow and long process [12]. Therefore, powerful and efficient domestic sewage chemistry is a primary approach to increase the biodegradability of pollutants, or as a more complex type of treatment to reduce COD or to minimize COD [13]. It is important to use technology to achieve high performance and low resource consumption [14]. To acheive high quality of water, it is very important to cnsider the consistency of the treatment process along with equipment [15]. Due to the continuous mechanical cleaning results due to the formation and decay of acoustic cavitation bubbles near the conductor surface, ultrasonic waves are used in combination with electrocoagulation or chemical decomposition methods to decontaminate conductors [16].

At present, various physicochemical processes such as chemical coagulation and biological flocculation reverse osmosis adsorption thrombolysis and biological processes constructed wetlands and other emerging technologies such as advanced oxidation processes practices and membrane technology have been adopted for the treatment of domestic wastewater [17]. The physicochemical process is not cost-effective, includes the overuse of chemicals, and produces large quantities of sludge. Simultaneously, the biological treatment method requires high dilution and it is a slow and time-consuming process. Hence, there is a need to search for a robust and cost-effective way to treat domestic wastewater. From the point of view of high performance and low resource usage, electrochemical technologies can be used either as a primary method to boost the biodegradability of the pollutant or as an advanced form of treatment to further reduce COD in wastewater to achieve appropriate effluent consistency [18]. The key advantages of the EC process compared to other traditional methods are simple experimental set-up and operation, less treatment time, no addition of chemicals, faster sedimentation of flocs and development of less sludge, and high efficiency in the removal of pollutants with lower electrical energy usage [15]. Electrocoagulation (EC) has been called the development of related chemistry since the last century [19]. It has been used in the past to treat various types of waste [20]. Since 1970, electrocoagulation has become increasingly common around the world for the treatment of commercial waste containing important metals [21]. Electrocoagulation offers great potential for removing soluble ionic species, a very important metal, from wastewater [22]. Electrocoagulation is one of the electrochemical processes in which soluble iron (Fe) and/or aluminum (Al) is used as the anode and/or cathode, and metal ions (Fe^2+^ or Fe^3+^, Al^3+^) are released due to anodic oxidation [23, 24].

Anode reaction:

(1) $$\begin{array}{c} \text{M}⟶{\text{Mn}}^{+}+{\text{ne}}^{-}. \end{array}$$

Cathode reaction:

(2) $$\begin{array}{c} {\text{nH}}_{2}\text{O}+{\text{ne}}^{-}⟶\frac{\text{n}}{2}{\text{H}}_{2}+{\text{nOH}}^{-}. \end{array}$$

The overall reaction:

(3) $$\begin{array}{c} {\text{Mn}}^{+}+{\text{nH}}_{2}\text{O}⟶\text{M}\left(\text{OH}\right)\text{n}+{\text{nH}}^{+}. \end{array}$$

In the overall reaction from reactions (1)–(3), the M (OH) n formed is used as a coagulant for the system. This can be aluminum hydroxide or iron hydroxide, depending on the electrodes used. Ultrasound (US) is transmitted to the material by waves that compress and decompress molecules [25, 26]. Cavitation bubbles are created when the negative pressure is large enough to disturb the distance between liquid molecules [27]. The collapse of these bubbles can produce very high temperatures and pressures, and these conditions can destroy the water molecules in the cavitation bubbles. Therefore, cleavage by ultrasonic decomposition of water molecules produces reactive OH percent radicals. It is a nonselective oxidizer for organic pollutants in wastewater [28]. This method can be achieved by combining higher reaction rates with pollutant decomposition and higher pollutant removal efficiency to improve the generation of radical ultrasound.

Based on the literature review, most of the previous studies focused on the efficiency of the electrocoagulation process for the removal of pollutants from the contaminated water and wastewater separately. This study aimed to compare and find the best highly effective and less power usage electrocoagulation process in removing COD from domestic wastewater to reduce the risks of pollutants on human and environmental health.

## 2. Materials and Methods

### 2.1. Sample Collection and Preservation Method

Samples were taken from the Jimma University cafeteria at the university's shared wastewater treatment plant in southwestern Ethiopia. Samples were collected in polyethylene (PE) containers, transported to the laboratory in 1 hour, and protected at + 4°C during the experiment.

### 2.2. Wastewater Characterization

The composition of the collected wastewater samples was determined in the laboratory and is shown in Table 1. Wastewater samples were taken from the cafeteria of the joint wastewater treatment plant at Jimma University in March 2021.

### 2.3. Experimental Setup

**Figure 1:**
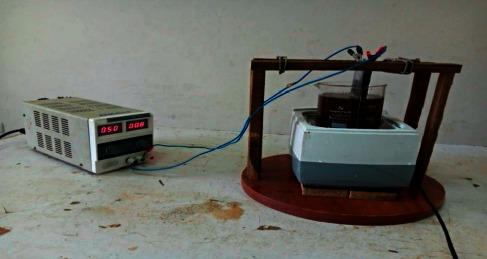
Schematic diagram of DCE and ACE process.

As indicated in Figure 1, the batch layout of the electrochemical reactor consists of 2.25 L of acrylic glass capacity, and the effective working capacity of wastewater is 1.0 L. The required COD concentration of wastewater was established by adding distilled water to the raw distilled effluent using a dilution factor. The initial pH interval 3–9, time interval 20–60, and current interval 0.2–0.5 value of the wastewater was measured with a pH meter (Elico: model LI120) and changed to the corresponding value in the range 3–9 with 0.1 NH_2_SO_4_ and 0.1 N NaOH solutions before the start of the test. Electrode combination (Al/Al): aluminum (Al) plates were used as anodes and/or cathodes with dimensions of 13 cm × 6 cm × 1 cm, respectively, in length, width, and thickness. The effective electrode surface area was 10 cm × 10 cm × 0.1 cm. A 2 cm gap between the bottom of the electrode and the bottom of the electrochemical cell reactor was maintained to allow proper agitation. The electrode distance between the anode and cathode changed by 2 cm. Before starting each experiment, the electrodes were washed with 15% HCl and distilled water. The anode and cathode were connected to the sonodirect and AC power packs (0–5 A, 0–270 V) in a unipolar parallel circuit. Samples were removed from the reactor at regular time intervals and centrifuged at 15,000 rpm for 15 minutes (REMI, model: R24) for COD and removal.

A batch reactor was also tested and a 1-liter wastewater sample was taken in a beaker for electrode combination. This EC process uses aluminum electrodes weighing 30.70 g and measuring 13 cm × 6 cm × 1 cm in length, width, and thickness. The copper wire is connected to a DC/AC power source and at one end is connected to the electrode by an electrical clip. The current was then supplied and the results were performed under various influence parameters. Eighty experiments were done in the laboratory and from eighty liters of wastewater sample twenty experiments were performed for direct current, twenty performed for alternative current, twenty for sonodirect current, and twenty for sonoalternative current. In those, all experiments COD was determined by considering different parameters like pH, current, and reaction time.

### 2.4. Response Surface Methodology (Design Expert11)

RSM is a mathematical-statistical method useful for optimizing chemical reactions and industrial processes and is often used in the design of experiments [29, 30]. Response surface methodology is a special set of mathematical and statistical methods, including the design of experiments, model fitting and validation, and state optimization. The purpose of RSM (Design Expert11) is to optimize the response of objects affected by a large number of variables. Response surface methodology (Design Expert11) is a useful statistical method for optimizing chemical reactions and industrial processes and is often used in the design of experiments. Response surface methodology (RSM) is the most common optimization method and is used in many areas, including the study of chemical and biochemical processes [31–35]. This technique is used to fit empirical models to experimental data [5, 36–39]. The RSM process is a group of statistical and mathematical methods used to develop and optimize processes in which the response surface of interest is affected by several variables [40–43]. RSM is a powerful technique with important applications in experimental design, new product development, and design, and optimization of existing product and process design [44, 45]. Define the impact of key factors alone or in combination with related processes.

### 2.5. Analysis

Removal efficiency (%) was measured based on the COD of domestic wastewater effluent before and after the Integrated SDCE and SACE process.

(4) $$\begin{array}{c} \text{COD removal }\left(\%\right)=\frac{Ci\;-\;Ct}{Ci}*100, \end{array}$$

where *Ci* and *Ct* are the COD (mg/L) of distillery wastewater before and after treatment, respectively.

(5) $$\begin{array}{c} \text{Power consumption}=\frac{VIt}{VR}\frac{\text{kwhr}}{{\text{m}}^{3}}, \end{array}$$

where *V* is cell voltage (Volt), *I* is applied current (amp), *t* is time (hr), and *VR* is volume of wastewater used (L).

## 3. Results and Discussion

### 3.1. Optimization with Response Surface Methodology (Design Expert11)

RSM is a mathematical-statistical method useful for optimizing chemical reactions and industrial processes and is often used in the design of experiments [17,46]. Response surface methodology is a special set of mathematical and statistical methods, including the design of experiments, model fitting and validation, and state optimization. The purpose of RSM (Design Expert11) is to optimize the response of objects affected by a large number of variables. Response surface methodology (Design Expert11) is a useful statistical method for optimizing chemical reactions and industrial processes and is often used in the design of experiments.

### 3.2. Removal Efficiency of COD

The removal efficiency (%) was measured based on the COD of domestic effluent before and after the integrated SDCE and SACE process. In Table 2, factors like pH, electric current, and reaction time were considered with different ranges. Similarly, the removal efficiency for COD was determined. Hence, using Al-Al electrode consumption by DC electrocoagulation, the removal efficiency is up to 82.6667%.

In Table 2, factors like pH, electric current, and reaction time were considered with different ranges. Similarly, the removal efficiency for COD was determined by considering all those factors. Hence, using DC electrocoagulation, the removal efficiency is up to 82.7% of COD with the power consumption of 24 Kwh/m^3^.

In Table 3, factors like pH, electric current, and reaction time were considered with different ranges. Similarly, the removal efficiency for COD was determined by considering all those factors. Hence, using AC electrocoagulation, the removal efficiency is up to 86.58% of COD with a power consumption of 21 Kwh/m^3^.

### 3.3. Optimization by SDCE and SACE

In Table 4, factors like pH, electric current, and reaction time were considered with different ranges. Similarly, the removal efficiency for COD was determined by considering all those factors. Hence, the results obtained by DC and AC are optimized by using SDC electrocoagulation and the removal efficiency is up to 88.6% of COD with a power consumption of 19 kwh/m^3^.

In Table 5, factors like pH, electric current, and reaction time were considered with different ranges. Similarly, the removal efficiency for COD was determined by considering all those factors. Hence, the results obtained by DC and AC are optimized by using SAC electrocoagulation and the removal efficiency is up to 92.35% of COD with a power consumption of 15 Kwh/m^3^. From the above tabulated data, it was investigated that sonoalternative current electrocoagulation consumes less power compared with sonodirect current electrocoagulation.

### 3.4. Al-Al Electrode Combination

In this experiment, two aluminum electrodes were combined in parallel to eliminate COD, taking into account various factors as per [47].

**Figure 2:**
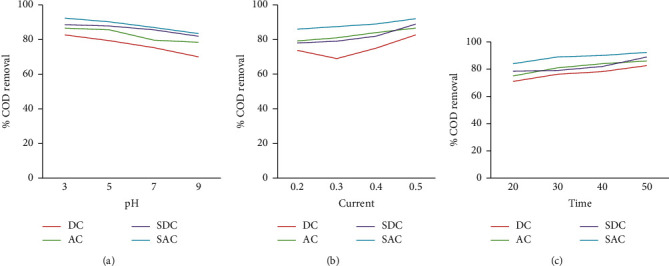
COD removal efficiency versus different factors (a) pH, (b) current, and (c) time, using Al-Al electrode.

**Figure 3:**
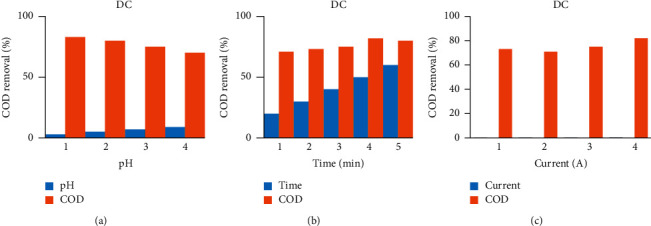
COD removal by a direct current with selected factors.

**Figure 6:**
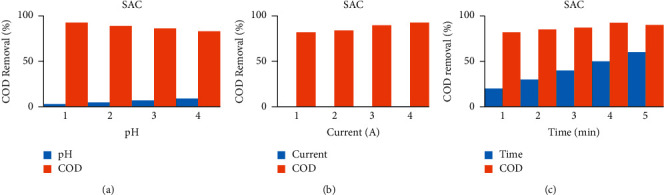
COD removal by a direct current with selected factors.

As shown in Figure 2, sonoalternative electrocoagulation (SACE) showed higher COD removal among the three factors of pH, current, and time. When pH increases from acidic media to basic media, the removal efficiency of the response decreases by all DCE, ACE, SDCE, and SACE as indicated in Figure 3 up to Figure 6. And also when current and time increase, the removal efficiency increases as indicated from Figure 3 up to Figure 6.

### 3.5. Operating Parameters for the Domestic Wastewater Treatment

Optimal maximal percentage COD removal with minimal electrical energy consumption is achieved by examining process parameters such as current, initial pH of wastewater, initial COD concentration, electrode spacing, and electrode combinations in the SACE process of domestic wastewater. The parameter conditions have been determined. The impact of these operating parameters on the SACE process is described below.

#### 3.5.1. Effect of Current

Current density is an important factor in controlling the ACE wastewater treatment process [26, 27]. The effect of current showed that COD removal increased from 86% to 92.5% as the current increased from 0.10 to 0.50 A/dm2. According to Faraday's law, the amount of electrochemically dissolved iron (Al) is proportional to the charge [48]. Therefore, as the current increases, the load increases, the generation of hydroxyl radicals increases, and pollutants are removed from the wastewater. Current must be maintained at optimal levels to avoid heat generation and excessive O_2_ generation at higher current and to achieve maximum COD removal with minimal power consumption.

#### 3.5.2. Effect of Initial pH Wastewater

The initial pH (pH 0) has a significant impact on the (SDC and SAC) electrocoagulation process. Under the conditions of different pH solutions, the allowable concentration of hydroxyl radical and the morphology of the aluminum hydroxide complex are different. The most preferred species under acidic conditions (pH < 5) are Al (OH) 2 +, Al (OH) +2, and Al (OH)_2_, which easily react with H_2_O_2_ to form OH. In the (pH = 3) solution, the maximum concentration of Al^2+^ is reached and the reaction of H_2_O_2_ produces more OH. In this experiment, the sample was pH adjusted with sulfuric acid solution and sodium. Hydroxides were up to pH of 9. These ranges show data on how acidic pH, neutral pH, and basic pH affect electrocoagulation efficiency in COD removal. However, the largest reduction was recorded at pH 3 (82.7%) by DC, (86.58%) by AC, (88.585) by SDC, and (92.35%) by SAC.

#### 3.5.3. Effect of Reaction Time

In particular, in this study, the reaction time was 1 hour, during which the removal efficiency was checked at various minute intervals with the initial value as the baseline. In this work, laboratory results showed that the reaction time of 1 hour was almost sufficient to remove the contaminants. Increase response time and increase the efficiency of removing pollutants from wastewater [49].

### 3.6. Statistical Analysis

The ANOVA was used to examine the significance of the impact of each factor on the response, where *Y*_*i*_ is the response variable, *β*_0_ is the model (regression) constant, *β*_*i*_ is the linear terms, *β*_*ii*_ are the squared terms (second-order), *β*_*ij*_ is the interaction terms, and *X*_*i*_ and *X*_*j*_ are independent equation (6) [50]. This experimental design was performed as a CCD consisting of 20 experiments for each method. The empirical model represented by a second-order polynomial regression is used to describe the system behavior calculated through the following equation:

(6) $$\begin{array}{c} Y_{i}=\beta_{0}+\sum_{i=1}^{4}\beta_{i}\cdot X_{i}+\sum_{i\leq j}^{4}\sum_{j}^{4}\beta_{ij}\cdot X_{i}\cdot X_{j}+\sum_{i=1}^{4}\beta_{ii}\cdot X_{i}^{2}+e. \end{array}$$

According to Table 6, the model is significant. That means all *p* values less than 0.0500 indicate the model terms are significant.

(7) $$\begin{array}{c} \text{COD removal}\left(\%\right)=75.0595-4.2886A\\ +0.613219B+0.900932C-0.555552AB-0.27277AC\\ -0.0509426BC-0.749057A^{2}-0.56374B^{2}-0.456059C^{2}. \end{array}$$

According to Table 7, the model is significant. That means all *p* values less than 0.0500 indicate the model terms are significant. In this case, A, B, C, BC, A^2^, C^2^ are significant model terms.

(8) $$\begin{array}{c} \text{COD removal}\left(\%\right)=79.9591-3.48698A\\ +1.37078B+0.710239C-0.0300148AB-0.124931AC\\ +1.03489BC-0.627884A^{2}-0.0475851B^{2}-0.553833C^{2}. \end{array}$$

According to Table 8, the model is significant. That means all *p* values less than 0.0500 indicate the model terms are significant. In this case A, C are significant model terms.

(9) $$\begin{array}{c} \text{COD removal}\left(\%\right)=83.3598-2.56923A\\ +1.12626B+1.23451C-0.446068AB+0.163127AC\\ +0.562812BC-0.353705A^{2}-0.869186B^{2}-0.24478C^{2}. \end{array}$$

According to Table 9, the model is significant. *p* values less than 0.0500 indicate model terms are significant. In this case, A, B, C, AB, AC, A^2^, B^2^, C^2^ are significant model terms:

(10) $$\begin{array}{c} \text{COD removal}\left(\%\right)=86.6929-3.29592A\\ +0.630435B+0.558774C-0.193618AB+0.151853AC\\ +0.0600054BC-0.473002A^{2}-0.269835B^{2}-0.240449C^{2}. \end{array}$$

### 3.7. Interactions of Different Parameters and Responses by DC, AC, ISDC, and ISAC

### 3.8. Comparison of SDCE and SACE Process

**Figure 7:**
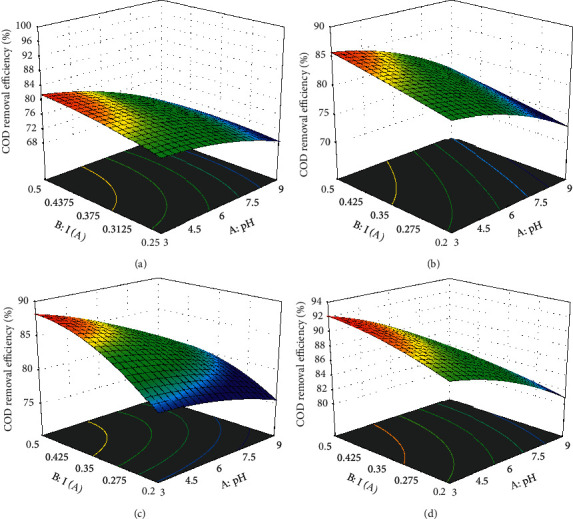
Three-dimensional response surface graphs for DC (a), AC (b), (c) ISDC, and (d) ISAC versus pH, time, and current.

An experiment was conducted to analyze the COD removal rate by comparing DCE, ACE, SDCE, and SACE methods using domestic wastewater. The results were optimized using the regression equation of RSM (Design Expert11) based on the central composite design. In the optimization of pH (A), current (B) and time(C) were selected as within range and the responses such as COD removal efficiency were maximized. For direct current electrocoagulation, the optimum value was obtained at pH 3, current 0.5 A, and time 50 min, such that the optimum value of COD was 82.6%. Similarly for alternative current electrocoagulation, the optimum value was obtained at pH = 3, current = 0.5 A, and time = 50 minutes such that the optimum value of COD was 86.6%. For sonodirect current electrocoagulation, the optimum value was obtained at pH of 3, current of 0.5 A, and time of 50 minutes. Such that the optimum value of COD removal was 88.5%. Similarly for sonoalternative current electrocoagulation, the optimum value was obtained at pH of 3, current of 0.5 A, and time of 50 minutes. Such that the optimum value of COD removal was 92.5%. The results are shown using operating conditions such as COD = 960 mg/L, wastewater Ph = 6.8, current density = 0.50 A, electrode spacing = 1 cm, electrode combination of Al-Al, and reaction time of 1 hour. From Figure 7 and the above finding results, it can be seen that the percentage of COD removal is higher in the ACE process than in the DCE process and higher in the SACE process than in the SDCE process. With ACE and SACE, sludge formation and impermeable layer formation are lower than with DCE and SDCE processes [51, 52]. Therefore, when comparing DCE and ACE and SDCE and SACE methods to remove the percentage of COD from domestic wastewater, the ACE method is more appropriate than using the DCE method and the SACE method is better than using the integrated SDCE procedure. From the ANOVA analysis, all the models presented from (6) to (10) for DC, AC, SDC, and SAC show as the model is valid. Because for all electrocoagulation methods provided, the value of P is less than 0.05 and it indicates that the model terms are significant. Results were obtained from samples taken and run in the laboratory based on the stated parameters.

## 4. Conclusion

This study demonstrated the application of direct current, alternate current, sonodirect current, and sonoalternate current electrocoagulation processes to the treatment of domestic wastewater.

The removal of COD from domestic wastewater was achieved with direct current, alternate current, sonodirect current, and sonoalternate current electrocoagulation being 82.6%, 86.58%, 88.6%, and 92.5%, respectively, under the optimal experimental conditions. From the finding, sonoalternate current electrocoagulation was more successful at removing % COD than the direct current, alternate current, and sonodirect current electrocoagulation methods. For direct current and sonodirect current, the formation of an impermeable oxide layer at the cathode and the occurrence of corrosion at the anode causes the COD removal process less efficiently compared with sonoalternate current electrocoagulation processes. From the ANOVA analysis, all the models presented from (6) to (10) for DC, AC, SDC, and SAC show as the model is valid. Because, in all methods, the value of P is less than 0.05, which indicates that the model terms are significant. From the experimental results it can be concluded that the sonoalternate current electrocoagulation has the lowest power consumption and higher process efficiency than the other electrocoagulation methods and can be a promising solution for removing pollutants from domestic wastewater.

## Figures and Tables

**Figure 4:**
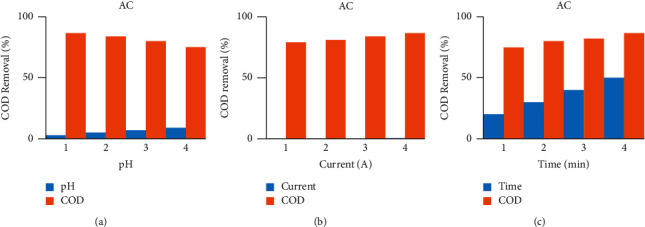
COD removal by alternative current with selected factors.

**Figure 5:**
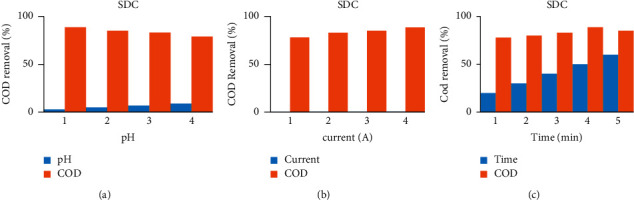
COD removal by sonodirect current with selected factors.

**Table 1:** Wastewater characterization.

No.	Parameters	Quantity	Unit
1	pH	6.8	—
2	Color	3	(%)
3	Turbidity	116	NTU
4	COD	960	mg/L
5	BOD	384	mg/L

**Table 2:** Input data and removal percentage of COD by DC electrocoagulation.

Run	Factor 1	Factor 2	Factor 3	Response	Power consumption, KWhr/m^3^
	A: pH	B: I (A)	C: time (minute)	COD removal efficiency (%)	
1	7	0.4	60	75.3333	12
2	5	0.4	40	78.5	10
3	9	0.5	50	70	16
4	3	0.5	50	82.6667	24
5	9	0.4	40	69.3333	14
6	5	0.4	40	78.6	16
7	7	0.4	40	75	14
8	9	0.5	30	68.9	23
9	3	0.5	30	80	25
10	5	0.4	40	79.87	19
11	3	0.3	30	76.2	17
12	7	0.4	20	71	12
13	5	0.2	40	73.8	21
14	7	0.4	40	74.8	17
15	9	0.3	30	69	18
16	7	0.4	40	75.1	16
17	3	0.3	50	79.2	18
18	9	0.4	40	69.3333	14
19	5	0.5	50	79.32	14
20	3	0.3	40	78.63	16

**Table 3:** Input data and removal percentage of COD by AC electrocoagulation.

Run	Factor 1	Factor 2	Factor 3	Response 1	Power consumption, KWhr/m^3^
	A: pH	B: I (A)	C: time (minute)	COD removal efficiency (%)	
1	7	0.4	60	79.6	8
2	5	0.4	40	84.69	7
3	9	0.5	50	78.4	11
4	3	0.5	50	86.58	21
5	9	0.4	40	75.36	10
6	5	0.3	30	82.36	12
7	7	0.4	40	79.312	13
8	9	0.5	30	75.692	20
9	3	0.5	30	83.521	21
10	5	0.4	40	82.69	17
11	3	0.3	30	81.658	15
12	7	0.4	20	75.368	11
13	5	0.2	40	79.23	19
14	7	0.4	40	79.321	12
15	9	0.3	30	75.25	15
16	7	0.4	40	79.2	14
17	3	0.3	50	82.36	15
18	9	0.4	40	75.36	11
19	5	0.5	50	85.56	12
20	3	0.3	40	83.25	14

**Table 4:** Optimization of COD removal efficiency by SDCE.

Run	Factor 1	Factor 2	Factor 3	Response 1	Power consumption, KWhr/m^3^
	A: pH	B: I (A)	C: time (minute)	COD removal efficiency (%)	
1	7	0.4	60	85.546	6
2	5	0.4	40	86.698	4
3	9	0.5	50	81.875	10
4	3	0.5	50	88.581	19
5	9	0.4	40	78.389	7
6	5	0.4	40	86.725	10
7	7	0.4	40	82.381	11
8	9	0.5	30	79.265	18
9	3	0.5	30	86.521	17
10	5	0.4	40	86.848	16
11	3	0.3	30	82.254	15
12	7	0.4	20	78.568	10
13	5	0.2	40	78.541	17
14	7	0.4	40	82.489	11
15	9	0.3	30	79.268	14
16	7	0.4	40	82.572	12
17	3	0.3	50	84.365	13
18	9	0.4	40	80.986	9
19	5	0.5	50	87.821	8
20	3	0.3	40	83.258	13

**Table 5:** Optimization of COD removal efficiency by SACE.

Run	Factor 1	Factor 2	Factor 3	Response 1	Power consumption, KWhr/m^3^
	A: pH	B: I (A)	C: time (minute)	COD removal efficiency (%)	
1	7	0.4	60	86.987	4
2	5	0.4	40	89.51	3
3	9	0.5	50	83.43	7
4	3	0.5	50	92.35	15
5	9	0.4	40	82.912	6
6	5	0.4	40	89.42	8
7	7	0.4	40	86.93	10
8	9	0.5	30	82.245	16
9	3	0.5	30	91.52	14
10	5	0.4	40	89.52	15
11	3	0.3	30	89.784	13
12	7	0.4	20	84.52	9
13	5	0.2	40	86.82	12
14	7	0.4	40	86.725	8
15	9	0.3	30	81.254	12
16	7	0.4	40	86.52	10
17	3	0.3	50	89.87	11
18	9	0.4	40	82.987	7
19	5	0.5	50	90.221	6
20	3	0.3	40	90.158	10

**Table 6:** ANOVA for the percentage of COD removal quadratic model by DC electrocoagulation.

Source	Sum of squares	df	Mean square	F-value	*p* value	
Model	358.33	9	39.81	93.12	<0.0001	Significant
A-pH	177.14	1	177.14	414.32	<0.0001	
B-I	2.25	1	2.25	5.27	0.0446	
C-time	9.57	1	9.57	22.39	0.0008	
AB	4.22	1	4.22	9.88	0.0104	
AC	1.20	1	1.20	2.81	0.01245	
BC	0.0175	1	0.0175	0.0410	0.00843	
A^2^	10.03	1	10.03	23.45	0.0007	
B^2^	3.78	1	3.78	8.84	0.0140	
C^2^	5.45	1	5.45	12.75	0.0051	
Residual	4.28	10	0.4275			
Lack of fit	3.06	5	0.6124	2.52	0.1663	Not significant
Pure error	1.21	5	0.2427			
Cor total	362.61	19				

**Table 7:** ANOVA for the percentage of COD removal quadratic model by AC electrocoagulation.

Source	Sum of squares	df	Mean square	F-value	*p* value	
Model	238.38	9	26.49	23.44	<0.0001	Significant
A-pH	116.06	1	116.06	102.73	<0.0001	
B-I	11.28	1	11.28	9.98	0.0102	
C-time	6.28	1	6.28	5.56	0.0401	
AB	0.0125	1	0.0125	0.0111	0.0184	
AC	0.2537	1	0.2537	0.2246	0.0457	
BC	8.11	1	8.11	7.18	0.0231	
A^2^	7.05	1	7.05	6.24	0.0316	
B^2^	0.0262	1	0.0262	0.0232	0.0820	
C^2^	7.74	1	7.74	6.85	0.0257	
Residual	11.30	10	1.13			
Lack of fit	9.29	6	1.55	3.08	0.1478	Not significant
Pure error	2.01	4	0.5023			
Cor total	249.68	19				

**Table 8:** ANOVA for the percentage of COD removal quadratic model by SDC electrocoagulation.

Source	Sum of squares	df	Mean square	F-value	*p* value	
Model	193.49	9	21.50	11.18	0.0004	Significant
A-pH	63.58	1	63.58	33.06	0.0002	
B-I	7.60	1	7.60	3.95	0.0348	
C-time	17.97	1	17.97	9.35	0.0121	
AB	2.72	1	2.72	1.42	0.0415	
AC	0.4300	1	0.4300	0.2237	0.0264	
BC	2.14	1	2.14	1.11	0.0162	
A^2^	2.24	1	2.24	1.16	0.0063	
B^2^	8.99	1	8.99	4.67	0.0559	
C^2^	1.57	1	1.57	0.8166	0.3874	
Residual	19.23	10	1.92			
Lack of fit	15.82	5	3.16	4.65	0.0585	Not significant
Pure error	3.40	5	0.6807			
Cor total	212.72	19				

**Table 9:** ANOVA for the percentage of COD removal quadratic model by SAC electrocoagulation.

Source	Sum of squares	df	Mean square	F-value	*p* value	
Model	210.62	9	23.40	840.61	<0.0001	Significant
A-pH	104.63	1	104.63	3758.18	<0.0001	
B-I	2.38	1	2.38	85.56	<0.0001	
C-time	3.68	1	3.68	132.25	<0.0001	
AB	0.5132	1	0.5132	18.43	0.0016	
AC	0.3726	1	0.3726	13.39	0.0044	
BC	0.0243	1	0.0243	0.8742	0.0218	
A^2^	4.00	1	4.00	143.59	<0.0001	
B^2^	0.8663	1	0.8663	31.12	0.0002	
C^2^	1.52	1	1.52	54.42	<0.0001	
Residual	0.2784	10	0.0278			
Lack of fit	0.1855	5	0.0371	2.00	0.2332	Not significant
Pure error	0.0929	5	0.0186			
Cor total	210.90	19				

## Data Availability

The data used to support the findings of this study is included in the article.

## Abbreviations

AC: Alternative current
BOD: Biological oxygen demand
COD: Chemical oxygen demand
CCD: Central composite design
DC: Direct current
EC: Electrocoagulation
FAS: Ferrous ammonium sulfate
IR: Infrared radiation
JUEEL: Jimma University Environmental Engineering Laboratory
JiT: Jimma Institute of Technology
JU: Jimma University
RSM: Response surface methodology
TDS: Total dissolved solids
TSS: Total suspended solids
SACE: Sonoalternative current electrocoagulation
SDCE: Sonodirect current electrocoagulation.
